# Beyond dual targeting: redefining the treatment horizon for true extramedullary myeloma? – a critical appraisal

**DOI:** 10.3389/fonc.2026.1822293

**Published:** 2026-04-15

**Authors:** Qinghua Ke, Shiqiong Zhou

**Affiliations:** Department of Chemoradiotherapy, The First People’s Hospital of Jingzhou, Jingzhou, Hubei, China

**Keywords:** bispecific antibodies, CAR - T therapy, critical appraisal, extramedullary myeloma, immunotherapy sequencing, talquetamab, teclistamab

## Abstract

The management of relapsed/refractory multiple myeloma with true extramedullary disease (EMD) remains a critical challenge. The phase 2 RedirecTT-1 study evaluated the combination of two bispecific antibodies, talquetamab (anti-GPRC5D) and teclistamab (anti-BCMA), in this high-risk population. While the reported overall response rate of 79% and 12-month progression−free survival of 61% are promising, a balanced interpretation requires caution. The single−arm design, short follow−up (12.6 months), and indirect comparisons with CAR−T therapy preclude definitive conclusions about comparative effectiveness. Notably, grade 3–4 infections occurred in 31% of patients, with five treatment−related deaths – a toxicity burden that may limit real−world implementation outside specialized centers. Responses were observed after prior BCMA−directed CAR−T therapy, but heterogeneity within this subgroup (time from CAR−T to progression ranging from 98 to 1030 days) tempers enthusiasm for universal sequencing strategies. Key unresolved questions include optimal sequencing with CAR−T, mechanisms of resistance, predictive biomarkers, and the feasibility of delivering this intensive regimen in routine practice. This commentary argues that dual bispecific therapy is a potent but still investigational option for EMD, and its definitive positioning requires randomized data and head−to−head comparisons.

## Introduction

The management of relapsed/refractory multiple myeloma (RRMM) with true extramedullary disease (EMD) – defined as plasmacytomas noncontiguous with bone – represents one of the most formidable challenges in contemporary hematology−oncology. Patients with this condition face uniformly poor outcomes with conventional therapies. In this context, Kumar and colleagues presented the phase 2 results of the RedirecTT-1 study, evaluating the combination of two T−cell−redirecting bispecific antibodies – talquetamab (anti−GPRC5D) and teclistamab (anti−BCMA) – exclusively in patients with drug−resistant, true EMD. The reported overall response rate of 79%, with 54% achieving complete response or better and a 12−month progression−free survival of 61%, appears impressive at first glance. However, these results must be interpreted with appropriate caution. As with any non−randomized study, the absence of a comparator arm, relatively short follow−up, and notable toxicity profile warrant a critical appraisal before positioning this combination as a new standard. This commentary aims to provide that balanced perspective.

## Contextualizing efficacy: the perils of indirect comparisons

The efficacy observed here has been compared to emerging real−world data. A recent multicenter analysis of 80 patients with true EMD treated with either CAR−T cells or bispecific antibody monotherapy reported response rates of 100% with cilta−cel and 82% with ide−cel, compared to 29−36% with single−agent bispecifics (1). While these cross−trial comparisons are interesting, they are inherently limited by differences in patient selection, prior therapies, and response criteria. The suggestion that the talquetamab−teclistamab combination “may bridge much of the efficacy gap” between single−agent bispecifics and CAR−T therapy is speculative without randomized evidence. We cannot conclude that dual bispecific therapy is superior to sequential monotherapy or non−inferior to CAR−T based on indirect comparisons alone. The 83% response rate among patients with prior BCMA−directed CAR−T therapy is noteworthy, but this subgroup comprised only 20% of the study population, and the median time from CAR−T to study entry was 295 days (range 98−1030 days). This wide range includes both patients with indolent post−CAR−T progression and those with aggressive relapse. Whether dual bispecifics are effective in the sickest patients – those progressing within weeks of CAR−T infusion – remains an open and critical question. Overinterpretation of this subgroup analysis risks overestimating the regimen’s utility in the most refractory settings.

## Therapeutic sequencing: unresolved questions for the clinician

For the practicing oncologist, the central question of therapeutic sequencing is far from settled based on this study. In an EMD patient presenting today, should we use dual bispecific therapy immediately, or reserve it for post−CAR−T relapse? The study’s inclusion of 20% prior CAR−T recipients provides partial but incomplete guidance. The authors note that the median time from CAR−T to study entry was 295 days. While this suggests a cohort with relatively indolent post−CAR−T progression, the reported range (98 to 1030 days) highlights substantial heterogeneity. This heterogeneity within the subgroup underscores that the efficacy of dual bispecifics in patients with ultra−early, aggressive relapse remains undefined. Conversely, for CAR−T−naïve patients, the 15.4−month median progression−free survival with this off−the−shelf combination raises the possibility that delaying CAR−T until after bispecific failure might preserve efficacy of both modalities. However, this hypothesis ignores potential mechanisms of resistance that could compromise subsequent CAR−T, such as T−cell exhaustion or antigen downregulation. Determining whether we can sequence immunotherapies to maximize cumulative benefit requires prospective randomized trials, not post−hoc subgroup analyses.

## Mechanisms of resistance and biomarkers of response: more questions than answers

The mechanisms underlying primary and acquired resistance to this combination warrant urgent investigation. Preclinical studies presented at the 2024 EHA Congress demonstrated a strong correlation between ex vivo sensitivity to teclistamab and talquetamab, suggesting shared determinants of response despite targeting different antigens (2). Patients with poor responses exhibited higher LDH levels, increased regulatory T−cell proportions, and lower effector−to−target ratios in the bone marrow microenvironment. While these associations are hypothesis−generating, they do not yet constitute validated biomarkers for clinical decision−making. T−cell dysfunction due to continuous activation appears to drive acquired resistance. This raises the possibility that treatment−free intervals or reduced−frequency dosing – such as the monthly maintenance schedule employed here for responding patients – may mitigate exhaustion and prolong response duration. The finding that 93% of patients who switched to monthly dosing maintained or deepened their responses supports this hypothesis, but this analysis is *post hoc* and subject to selection bias – patients who remained on monthly dosing were likely those already responding well. Without randomized dose−reduction or discontinuation studies, we cannot separate the effect of dosing schedule from patient selection.

## Managing toxicity: the critical role of supportive care – and its real−world barriers

The toxicity profile of this combination demands sophisticated supportive care infrastructure. Grade 3−4 infections occurred in 31% of patients, and five treatment−related deaths were attributed to infections – a sobering reminder that efficacy gains come at the cost of profound immunosuppression. The phase 1 RedirecTT-1 report documented grade 3−4 infections in 64% of patients, suggesting that the lower rate in this phase 2 study may reflect improved infection prevention strategies, particularly the higher utilization of intravenous immunoglobulin (IVIG) (87% vs. 57%) (3). However, the median time to first IVIG administration of 50 days means many patients remained at risk for infections during the first seven weeks of therapy. Emerging consensus recommends primary IVIG prophylaxis for all patients receiving bispecific therapy, regardless of baseline IgG levels, as IgG measurements in MM are confounded by monoclonal protein (4). Yet this recommendation is not yet evidence−based; ongoing studies comparing universal versus IgG−guided IVIG will be crucial. Moreover, the original commentary glosses over the practical barriers inherent in global and real−world practice: variable access to IVIG, reimbursement pathways, logistical coordination, and the need for specialized infectious disease support. The treatment−related mortality rate of approximately 4% (estimated from the phase 2 cohort) may be higher in less experienced centers. A more balanced discussion would acknowledge that these toxicities could limit generalizability and that patient selection is critical.

## Discussion and future directions: what is needed before clinical adoption?

Several limitations of the RedirecTT-1 study must be critically interrogated before this combination can be considered a standard of care. The relatively short follow−up (12.6 months) precludes assessment of very−long−term outcomes, late toxicities, or secondary malignancies. The exclusion of patients with central nervous system involvement or plasma cell leukemia leaves the efficacy in these highest−risk subgroups undefined. Moreover, the study’s single−arm design cannot establish comparative effectiveness against sequential bispecific monotherapy or CAR−T therapy (5). The ongoing phase 3 MonumenTAL−6 trial (NCT06208150) will provide higher−level evidence, but it will not directly address the CAR−T versus dual bispecific question. To responsibly advance this field, several specific questions must be addressed:

1. Can baseline or dynamic biomarkers prospectively differentiate between patients most likely to benefit from dual bispecifics versus those who should proceed directly to CAR−T? (This remains unproven).
2. Is a universal IVIG prophylaxis strategy superior to an IgG−guided strategy in preventing grade ≥3 infections during the first six months of therapy? (Randomized trials are needed).
3. How does the quality of life and treatment burden of continuous dual bispecific therapy compare to a one−time CAR−T infusion with a treatment−free period? (Patient−reported outcomes are absent from the current study).
4. What is the real−world generalizability of these results outside academic centers? (Registry data are urgently required).

Until such evidence accumulates, dual bispecific therapy should be considered a potent but still investigational option for EMD – preferably used in clinical trials or at experienced centers with robust supportive care, rather than as a new universal standard.

## Conclusion

For the hematologist confronting a patient with progressive EMD tomorrow, the message from RedirecTT-1 is promising but not definitive. Dual targeting of GPRC5D and BCMA with talquetamab plus teclistamab offers a potentially powerful option that is immediately available. However, the current evidence is insufficient to position this combination as a transformative or preferred therapy over existing options such as CAR−T or sequential bispecific monotherapy. The single−arm design, short follow−up, indirect comparisons, and notable toxicity profile – including five treatment−related deaths – demand a cautious, balanced interpretation. The path forward lies in refining patient selection through validated biomarkers, optimizing supportive care with evidence−based IVIG protocols, and – most importantly – generating randomized data to define the optimal sequence among our expanding armamentarium of T−cell−redirecting therapies. This study provides an important signal of activity for EMD but should be seen as a starting point, not a final answer.
